# Institutional framework for integrated Pharmaceutical Benefits Management: results from a systematic review

**DOI:** 10.5334/ijic.2253

**Published:** 2015-09-29

**Authors:** Tomasz Roman Hermanowski, Aleksandra Krystyna Drozdowska, Marta Kowalczyk

**Affiliations:** Head of Department of Pharmacoeconomics, InterQuality Project Leader (7FP), President of the Polish Society of Health Economics, Medical University of Warsaw, Warsaw, Poland; Medical University of Warsaw, Warsaw, Poland; Medical University of Warsaw, Warsaw, Poland

**Keywords:** Pharmaceutical Benefits Management, Pharmacy Benefit Managers, generic substitution, generic drugs, online adjudication of pharmacy claims, e-Prescribing, integrated care

## Abstract

**Objectives:**

In this paper, we emphasised that effective management of health plans beneficiaries access to reimbursed medicines requires proper institutional set-up. The main objective was to identify and recommend an institutional framework of integrated pharmaceutical care providing effective, safe and equitable access to medicines.

**Method:**

The institutional framework of drug policy was derived on the basis of publications obtained by systematic reviews. A comparative analysis concerning adaptation of coordinated pharmaceutical care services in the USA, the UK, Poland, Italy, Denmark and Germany was performed.

**Results:**

While most European Union Member States promote the implementation of selected e-Health tools, like e-Prescribing, these efforts do not necessarily implement an integrated package. There is no single agent who would manage an insured patients’ access to medicines and health care in a coordinated manner, thereby increasing the efficiency and safety of drug policy. More attention should be paid by European Union Member States as to how to integrate various e-Health tools to enhance benefits to both individuals and societies. One solution could be to implement an integrated “pharmacy benefit management” model, which is well established in the USA and Canada and provides an integrated package of cost-containment methods, implemented within a transparent institutional framework and powered by strong motivation of the agent.

## Background and problem statement

In the framework of the InterQuality research project [1], the authors of this paper used the assumptions of new institutional economics to examine and assess the functioning of institutions, to verify their objectives and efficiency in resources management, generation of transaction costs, as well as to analyse institutional environment, including the political system, and efficiency of judicial law enforcement systems (based on the degree of court judgement enforceability, effective law enforcement measures, etc.). Within the new institutionalism, the agency theory (including the principal-agent model) was used to describe business relationships between payers and service providers in the health sector, and to analyse models of incentive systems. The principal-agent problem is based on information asymmetry where one party of an economic relationship has information advantage over the other party in terms of knowledge of the transacted service or product. It is a major challenge which may have a distortionary effect on the operation of the market. According to the principal-agent theory, difficulties in enforcing the quality of services in health care are related to the fact that it is not possible to work out an agreement that would cover all important aspects of the agent's (service provider) activity, and the objectives of the principal (payer) are usually multidimensional and can defy measurement.

Access to health care that meets a specific quality threshold is recognised as the primary goal for all aspects of health care – for pharmaceutical care as well as care provided at hospitals, by physicians and via other health care providers [2]. Thus, pharmaceutical care reimbursement, as with other aspects of health care reimbursement, should work in tandem and be well aligned with pharmaceutical care delivery systems to support satisfactory levels of patient access. The reimbursement as well as the delivery of pharmaceuticals should reflect the patients’ right of access to appropriate medicines and, to the extent possible, enhance patient access to appropriate medicines in a timely manner.

The market structures in Europe differ significantly, which lead to differences in terms of transparency of drug policy (which should be understood as all organisational and legal activities through which citizens are provided access to safe, effective pharmaceutical care, while reducing patient participation in treatment costs) [3]. Different institutional settings are indirectly translated into different outcomes of the health care systems. For example, in Germany, the potential cost-saving generated by generic substitution was transmitted into incentives for doctors and patients (decision-makers) more effectively than in the UK. The lack of mechanisms which would leverage physician agency in the UK results in more costly prescribing patterns and decreases possible savings for health care budgets which could be otherwise generated by effective adoption of low-cost treatment [4].

Generic policy in Germany and the UK can be analysed from the perspective of the principal-agent theory. Both in Germany and the UK, control on prescribing at the physician level is weak and the financial incentives for doctors are limited. In the UK, doctors “have weak knowledge of the prices of some of the most widely-prescribed medicines” and not always act as agents protecting the patients interests [5]. Moreover, doctors are not obliged to switch patients to less expensive medication as prescribing guidelines by National Institute for Clinical Excellence are not binding [4].

German doctors are more effective agents for their patients because of the impact of medicine choice on patients’ cost-sharing. In Germany, patients are liable for copayments, and those copayments are transferred to the sickness funds (a copayment of 10% of the drug sales price applies, along with an additional fee between 5 and 10 euros per prescription). Physicians are required to inform patients if the price of the prescribed medicine exceeds the reference price and therefore increases patients’ cost-sharing [4]. Hence, doctors act as price responsive agents for their patients who are liable to co-pay.

The UK National Health Service uses different pricing schemes for branded and generic drugs. Reimbursement rates for generic drugs under the Drugs Tariff are based on average ex-factory prices across available generic drugs in the UK. The average drug price at which the pharmacy is settled with National Institute for Clinical Excellence is determined, but the pharmacy can purchase drugs at lower costs from manufacturers. For that reason, the average price of generic drugs in the UK goes down faster than in Germany. Since the amount paid to a pharmacy is not related to the pharmacy's actual cost, this has led to a large decrease in generic drugs prices: in 2005, the average annual decline in generic prices in the UK was almost 5 times higher than in Germany. Overall, patients in the UK pay only 5.6% of drug costs [4].

During our research, we sought to identify the most effective financing models of drug policies and the institutional set-up necessary for their effective implementation. We have paid special attention to the Pharmacy Benefit Managers (operating in the USA and Canada), third-party administrators of prescription drug programmes. Pharmaceutical Benefits Management companies are private organisations which compete by offering their customers a variety of specialised clinically based and administrative tools, helping them manage drug expenditures by enhancing price competition and increasing medication cost-effectiveness.

### A closer look at the Pharmacy Benefit Manager business model. Pharmacy Benefit Managers market and benefits for the health care system

**Figure 1.**
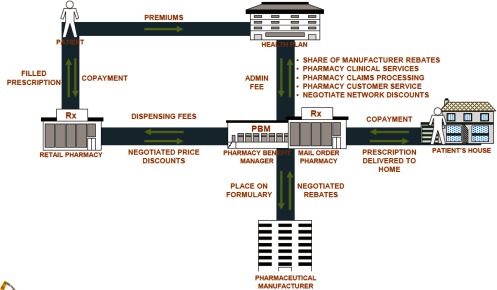
How does a Pharmacy Benefit Managers work – high-level value chain [6].

The Pharmacy Benefit Manager (in the USA and Canada) is a third-party administrator of prescription drug programmes. Pharmaceutical Benefits Management companies are private organisations. Pharmacy Benefit Managers’ administrative costs may be reimbursed through a direct fee or their costs may be subtracted from the rebates they receive from pharmaceutical companies before sharing them with clients. Pharmacy Benefit Manager clients include union-sponsored plans, small and large businesses, health plans, state and federal employee benefit plans, and government-funded Medicare and Medicaid plans. Referring to the principal-agent theory, Pharmacy Benefit Managers act as agents of their clients. Pharmacy Benefit Managers compete by offering their clients a range of tools and techniques that can help improve the quality and cost-effectiveness of the drug benefit (Figure 1).

A brief history of Pharmacy Benefit Managers in the USA is as follows: since 1960s, under pressure from trade unions, drugs have been included into insurance coverage (patients used to pay out-of-pocket for drugs dispensed by community pharmacies). This new task proved very difficult because it required quite a lot of new competences and specific infrastructure to be developed. Unprepared to face the new reality, insurers outsourced this function to a special type of institution that has emerged – the Pharmacy Benefit Managers. At the beginning, Pharmacy Benefit Managers were responsible only for administrative settlement of reimbursement claims; later, Pharmacy Benefit Managers added a number of other services (which are discussed in detail later). PCS was the name of the first Pharmacy Benefit Management set up in 1969. The rising sales and profits of these institutions allowed them to make investments in information technology. In 1980s, because of the rapid development of organisations such as Health Maintenance Organizations and increasing medication costs, Pharmacy Benefit Managers expanded the scope of service to include management of insurers’ drug policies, including a movement towards the better management and more optimal use of medications by care recipients’.

In the USA, there are about 50 Pharmacy Benefit Managers [7] and they process about 70% of prescriptions [8]. The Pharmacy Benefit Manager market in the USA has been very consolidated – in 2011, the market share of top 10 Pharmacy Benefit Managers was about 85% (in terms of annual prescription volume) [9]. Since then, the market consolidation has increased even further, especially after 2012, when Express Scripts acquired Medco Health Solutions for over $29 billion and increased its market share to 32% [10]. Pharmacy Benefit Managers may operate as independent entities. Some Pharmacy Benefit Managers are subsidiaries of health plans, others belong to large supermarket chains or pharmacy networks (CVS Caremark, Kroger Prescription Plans). Some large insurers, such as Cigna, offer in-house Pharmacy Benefit Manager functions. A number of smaller private Pharmacy Benefit Managers also operate.

In 2008, as much as 90% and 76% of drug spending by Medicare and non-Medicare population, respectively was covered by private plans in which the pharmacy benefit is managed by Pharmacy Benefit Managers. Approximately 82% of US prescription drug spending ($204 billion) was managed by Pharmacy Benefit Managers in 2008. Estimated Pharmacy Benefit Manager Savings (in the USA) were as follow:

- Reduction of prescription drug costs by 29% (on average);
- Reduction of US national prescription drug benefit costs by $85 billion (included $43 billion of cost savings for Medicare Part D) in 2008;
- Total estimated savings (between 2008 and 2017) will amount to about $1.3 trillion (included $693 billion of cost savings for Medicare Part D);
- Pharmacy management slowed the growth of prescription drug spending (from about 18% (1999) to 5.8% (2005) per year.

The main Pharmacy Benefit Managers tools and techniques are as follows [11]:

- Electronic claims processing (a real-time, point-of-sale system linked to pharmacies and distribution centres, which allows verification of coverage, drug interactions, formulary restrictions, etc.);
- e-Prescribing (prescriptions can be written, transmitted and filled electronically rather than manually);
- Formulary development and management (a list of prescription drugs approved for reimbursement by the plan sponsor contracting with a Pharmacy Benefit Manager);
- Networks of retail pharmacies;
- Generic substitution (dispensing of a chemically equivalent but less costly medicine instead of a brand name medicine that has an expired patent);
- Rebates and discounts (for the inclusion of manufacturer drug on a formulary at a preferred level);
- Therapeutic interchange (Pharmacy Benefit Managers may contact the prescribing physician and suggest that the physician authorise interchange of the original drug to the preferred one);
- Mail-service pharmacies that supply home-delivered prescriptions;
- Drug Utilization Review (assures patients receive appropriate drug therapy based on current medical guidelines);
- Disease management – Pharmacy Benefit Managers hire physicians and nurses to provide disease management services;
- Consumer information;
- Consumer compliance programmes.

### Agent's motivation to improve quality and lower costs by Pharmacy Benefit Managers instrument's application

The cost issue is particularly important in the USA as health care expenditures have been increasing from year to year to exceeded 17.4% of gross domestic product in 2009 [12]. Expenditure on medicines accounts for as much as 60% of all medical expenses incurred directly by patients (the proportion is even higher among elderly). In response to that, some very effective initiatives have been taken in the USA to reduce treatment costs. Managed pharmaceutical care, in the form of Pharmacy Benefit Managers, have thus been an effective tool for quality improvement and cost reductions in the US context.

The results of our study indicate that the introduction of independent institutions – such as Pharmacy Benefit Managers in the USA – to coordinate functions associated with pharmaceutical care/drug policy has led to significant savings and increased quality of health care. In Europe, functions similar to those performed by Pharmacy Benefit Managers have been fulfilled by different organisations; however, cooperation and coordination among them have been largely insufficient. As a rule, there exists no separate entity which would manage the whole pharmaceutical care process in a coordinated manner, thereby increasing the efficiency of drug policy.

## Methods

We performed a comparative analysis of different pharmaceutical care tools typically used by Pharmacy Benefit Managers in the USA and now implemented in different institutional set-ups in the UK, the USA, Poland, Italy, Germany, and Denmark. The institutional framework was described on the basis of publications obtained from a systematic review. The review was conducted in Medline, using a search strategy containing words connected to Pharmacy Benefit Manager tools and countries of interest.

Inclusion criteria were as follows: (1) articles concerning Pharmacy Benefit Management, electronic prescribing, real-time claims adjudication, generic substitution and integrated pharmaceutical care experiences in the USA, UK, Poland, Italy, Denmark and Germany, (2) descriptive articles, analysing development, structure and/or impact of pharmaceutical care tools on health care system, (3) publications from the last 10 years (the cut-off date of the review was 20 February 2014), (4) publication language: English or Polish. Accordingly, exclusion criteria were: (1) articles describing topic other than described above, (2) articles that do not specify the country or concern a country other than those of interest, (3) articles that do not contain any quantitative measures or qualitative data, specifying the real process of Pharmacy Benefit Manager tools development or usage, (4) experiences from countries other than listed above, (5) publications more than 10 years old.

**Figure 2.**
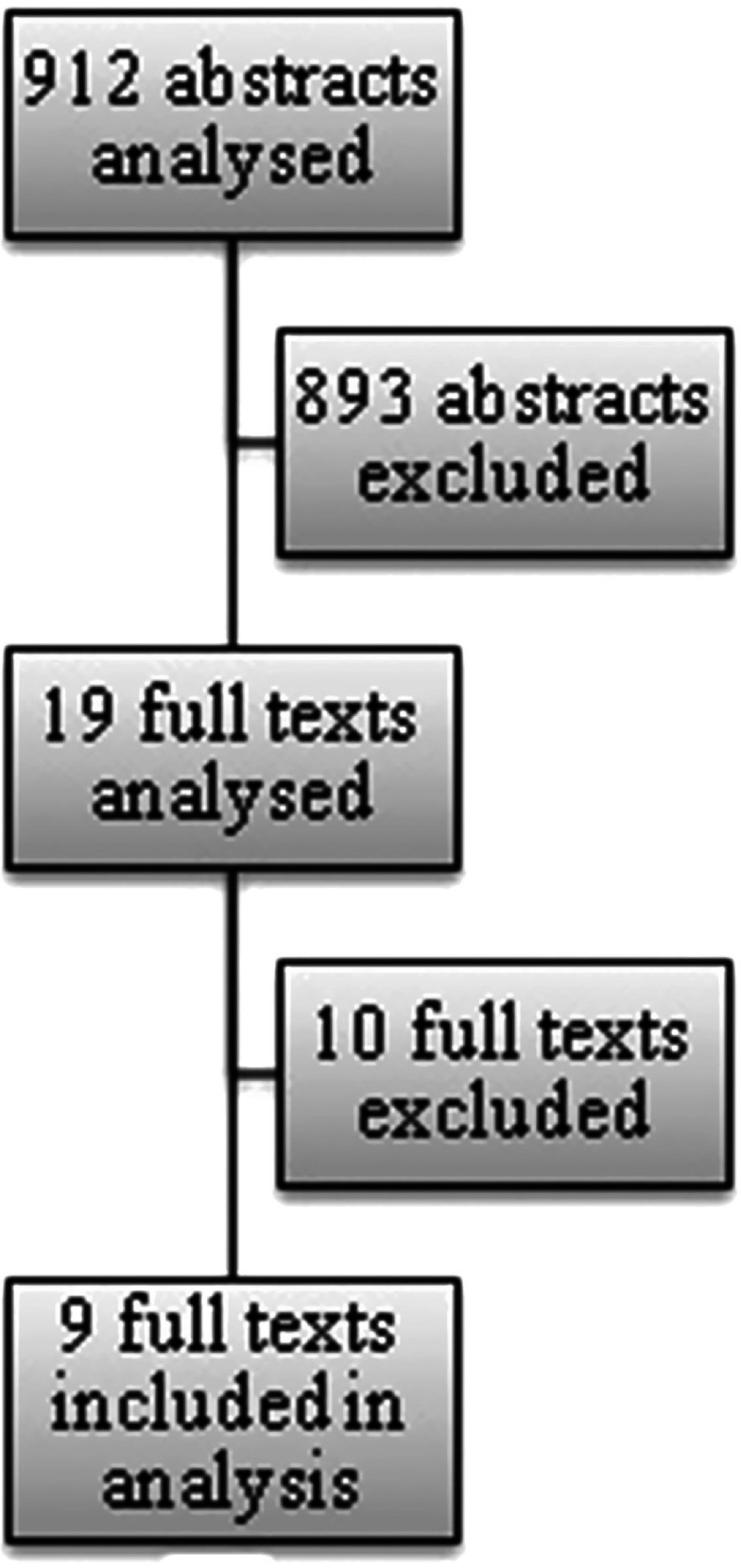
Abstracts and full texts selection process.

In the first phase (titles and abstracts selection) 29 of 912 abstracts were chosen. The next stage of review (full texts selection) resulted in the final choice of 9 publications. The selection process is illustrated in Figure 2.

Additionally, manual search for monographs and websites providing more information was performed by researchers, using the same inclusion and exclusion criteria, and 39 sources found this way were included in the study, with citations listed in the references section.

## Results

### United States of America

According to research by the Centers for Medicare and Medicaid Services, Pharmacy Benefit Manager tools contributed to the containment of the growing prescription drug expenditure from 5.3% in 2009 to 3.5% in 2010 [13]. Through promoting the use of generic drugs, Pharmacy Benefit Manager tools lower the costs for payers and consumers. The most prominent tool was the tiered copayment that moved medication prescription and administration towards less expensive generic drugs [9]. According to a study by Express Scripts from October 2005, American consumers could have saved $20 billion in 2004 through the use of generic drugs [14].

Pharmacy Benefit Managers pioneered the use of revolutionary e-Prescribing technology and contributed to the adoption of e-Prescribing in Medicare [15]. Electronic prescription systems can be understood as a solution that eliminates traditional prescription writing from health care services. The system consists of three separate services – Decision Support, Electronic Medical Records and Electronic Transmission of Prescriptions. In the USA, the Health Information Technology for Economic and Clinical Health Act promotes implementation of this cutting-edge technology since e-Prescribing is considered an important usage of electronic medical records [16]. Standards for issuing, recording and transmitting prescriptions have been established by the National Council for Prescription Drug programmes. The SCRIPT standard is of particular importance as it describes data formats. e-Prescribing technology in the health care market [17] seems most advanced technology in the USA.

The Centers for Medicare & Medicaid Services started an incentive programme for e-Prescribing, under which eligible physicians who successfully used electronic were entitled to receive an incentive payment of 1.0% in 2011 and 2012, and 0.5% in 2013. What is more, there were also penalties for those who did not e-Prescribe – 1.0% in 2012, 1.5% in 2013, and 2.0% in 2014 and beyond [18]. This programme was a success as demonstrated by e-prescription frequency increasing significantly [19].

Electronic prescribing systems in the USA are expected to generate savings of about 29 billion dollars as a result of fewer prescription duplications, more generic prescriptions, elimination of drug–drug interactions and dosing errors, and other medicine-related problems and the resulting decreased number of consultations with physicians or inpatient hospital days [20].The basic services, such as on-line claims adjudication, are now offered by all Pharmacy Benefit Managers. Unlike the banking and other market sectors where business transactions take only seconds to complete, the health care sector is far more inefficient in transaction processing. This scenario could be changed with the adoption of real-time claims processing. Successful reforming of Claims Processing with Real-Time Adjudication in the USA has reduced the time spent on administrative activities and excess paperwork [21].

Real-time adjudication of pharmacy claims is becoming increasingly recognised as a valuable tool among health care providers. Nowadays, a fully functional system of real-time adjudication operates both in the USA and New Zealand. European Union member countries have yet to implement this progressive technology. For this system to work, payers would have to support it on a large scale in Europe. Payers would have to introduce software that could process claims in a matter of seconds. Nowadays, with the advanced software technology ready available, the real issue is the attitude of providers towards innovations rather than the absence of suitable technical solutions.

Formulary is a list of prescription drugs chosen for their clinical and cost-effectiveness and is used to determine plan coverage and patient cost share. It is also known as a preferred products list. Formularies serve Pharmacy Benefit Managers to “channel” patients to a particular product and to point out an apparent “best value” among many drugs of therapeutic category. Patients are offered financial incentives (e.g. lower co-payments) to buy drugs from a formulary. This has helped Pharmacy Benefit Managers increase purchase volume of the drugs and maximise rebates from drug manufacturers [8]. Pharmacy Benefit Managers typically use panels of independent clinical experts to create lists of active substances accepted for reimbursement in order to support clinically correct and cost-effective prescribing. Such classification is called “tiering”. Tiered formularies determine co-payment structure and cost-sharing. Placing drugs within particular copayment tiers depends on their safety, effectiveness, efficacy as well as direct costs and discounts that payers can negotiate with drug manufacturers. Greater use of generic medications could result in important health care savings while maintaining quality of care [22–24]. In Europe, one of the main objectives of health policy is generic substitution. Nevertheless, in some countries the share of generics is still relatively low. Substituting generics for patented drugs appears to be not good enough for Pharmacy Benefit Managers. It is taken for granted that, after patent expiration, generics are substituted for original drugs. In the USA, this happens in a matter of weeks rather than months or years (as is the case in Europe). The ultimate objective is to reduce the price of generic drugs as much as possible.

The instrument used for this purpose is managed competition between generics manufacturers. Pharmacy Benefit Manager selects, often by a public tender advertised on the Internet, one or two manufactures who offer the biggest rebates. In exchange, their drugs are included in the first tier of the formulary, without any or with a nominal co-payment. The purpose of the Pharmacy Benefit Manager contracts with pharmaceutical manufactures is also to promote the manufacturers’ drugs to patients and physicians [25]. Thanks to Pharmacy Benefit Manager drug promotion, the manufacturer of a preferred drug may reduce marketing costs, sharing the achieved savings with the Pharmacy Benefit Managers, who passes a part of them to Pharmacy Benefit Manager clients, who in turn may reduce the insurance premiums. It is the patient who is the final beneficiary, paying less for his drug benefit. Generics classified as non-preferred may be reimbursed only in medically justified cases. They are usually dispensed against a higher, second-tier co-payment. The third group of the most expensive drugs may be dispensed only upon prior approval by the Pharmacy Benefit Managers. However, Pharmacy Benefit Manager clients are the ones who make the final choice of drugs listed on the formulary [26]. Pharmacy Benefit Managers are also able to implement efficient solutions to the epidemic of prescription drug fraud, waste and abuse [27]. Prescription drug diversion is both a public health threat and a significant cost driver.

### Denmark

Electronic prescribing has been successfully implemented in Denmark. Currently, almost all physicians routinely prescribe medicines electronically with e-Prescribing. About 97% of general practitioner practices declared to have been using e-Prescribing regularly, therefore Denmark may be recognised as one of the leading countries, if not the leading European country, implementing this cutting-edge technology. However, practitioners still can choose their preferred way of issuing prescriptions: it may be done by writing, electronically, via telephone or fax. The Danish structure for e-Prescribing process consists of a Medicine Profile and a Prescription Server which have been introduced in recent years. The electronic Medicine Profile provides for the monitoring of prescription drug purchasing in Denmark. All purchases are registered automatically and accumulated in individual patients’ medical profiles. In 2007, the Danish Medicines Agency established a central prescription server which provides the option of electronic transfer of prescriptions from physicians to pharmacies. After the successful large-scale implementation of e-Prescribing in Denmark, Computer Sciences Corporation declared their readiness to do the same across Europe [28].

In Denmark, the reimbursement decisions are the responsibility of the Danish Medicines Agency. Whenever reimbursement of pharmaceuticals under a health insurance is involved, the Reimbursement Committee is consulted. The Committee consists of up to seven individuals, at least two of them working as general practitioners. Members are recruited for a four-year term by the Minister of Health and Prevention from among experts recommended by the Agency. One member represents a third-party payer. The Danish Medicines Agency convenes a Committee Meeting once a month, generally to discuss the reimbursement status of pharmaceuticals. The Committee is asked for an advice when a company has applied for general reimbursement of a new pharmaceutical and takes part in choosing the criteria for different kinds of individual reimbursement [29].

Generic substitution has been used in Denmark since 1991. Decisions about which generic products are appropriate for substitution are taken by the Danish Health and Medicines Authority. The National Health Service organises the reimbursement in a way to incentivize generic substitution and the general rule that only the lowest cost products are financed is applied. Pharmacies are obliged to dispense these lowest cost products to patients, unless a doctor or a patient decides otherwise [30].

The real-time claims adjudication has been implemented in Denmark at the Central Reimbursement Register [31].

### United Kingdom

The Electronic Prescription Service enables primary care prescribers to create and transfer electronic prescriptions using a computer system. The e-Prescribing process has several stages: first, such prescription is transmitted to the Electronic Prescription Service. Then it can be downloaded by a pharmacy whose computer system has been upgraded to use Electronic Prescription Service. Patients can choose a dispensing contractor to retrieve the electronic prescription automatically – a paper form is no longer necessary. Electronic Prescription Service is being implemented across the UK in primary care settings in two stages, to ensure that commitments are met. These settings consist of community pharmacies, general practitioner practices and dispensing appliance contractors.

An extensive literature search confirmed that real-time adjudications have not yet been fully implemented in the UK. However, online adjudication of reimbursement claims for medications and medical devices supplied will be prospectively available for dispensers [32].

In the UK, the Department of Health is responsible for deciding on drug reimbursement. The crucial criteria in determining whether to cover the treatment are cost and cost-effectiveness ratios. Across the UK, decision-making has been increasingly devolved to the four home nations of England, Scotland, Wales and Northern Ireland. Hence, the National Health Service in each devolved country makes decisions at the country level, although stakeholders may influence the decision-making process. The pharmaceutical industry, patients, pharmacists, doctors and the National Institute for Clinical Excellence are involved. Since January 2002, all drugs and treatments approved by National Institute for Clinical Excellence are automatically financed by the National Health Service in England [33].

The National Health Service supports generic substitution through a special policy towards physicians. General practitioners are allowed to reinvest their savings (in their practice) if they spend below their budget. In this way, physicians are encouraged to prescribe generics [34], although they can still tick a special box on the prescription to insist on the branded medicine [35]. Being able to reinvest savings may be a fairly good incentive for a physician who is ready to monitor changing drug prices. Dispensing generics is also profitable for pharmacists as they obtain a refund on the list price plus a flat fee per drug sold. Therefore, if they manage to acquire a drug below the list price, they hold a higher margin [34]. Generic substitution is also promoted by the National Institute for Health and Clinical Excellence, performing cost–benefit assessment of drugs and assessing the efficiency of the product in relation to its originator equivalent.

### Poland

Under the Act establishing the organisation and operation of a health care information system in Poland (2011), the widespread launch of electronic medical documentation was scheduled to take place on 1 August 2014 [36, 37]. All health care facilities were to keep and handle medical documentation in an electronic form instead of a paper form. Computer-generated prescriptions were expected to replace traditional ones and were supposed to be automatically assigned a unique ID number by the Healthcare Information Systems Center. However, only 10% of Polish hospitals currently declare their readiness to switch to this system.

Moreover, as a rule, doctor's offices in Poland do not have appropriate information technology equipment to be able to handle this task, in contrast to pharmacies. Nonetheless, there is apparently no intention to implement online adjudication of pharmacy claims which, in logical terms, could be the next step leading to substantial savings in this area. There is no or little understanding that the implementation of any e-Health technology would be much easier if it begins at the pharmacy level.

Electronic prescription is one of the priorities of the health care computerisation process currently taking place in Poland [38]. According to preliminary assumptions, the project was scheduled to be completed by January 2012. Now experts predict that from 2020 onwards, every Polish patient will have an electronic health record and e-prescriptions will be widely used. A prototype of the e-prescription system has been implemented by the Center for Health Information Systems [39], in the framework of the P1 project: Electronic Platform Collection, Analysis and Dissemination of Digital Resources for Medical Events. The Center for Health Information Systems is managed by the Ministry of Health. An Electronic Platform will be connected to computer systems used by the National Health Fund [39].

From March 2011, a trial implementation of e-prescription has been carried out in Leszno, which is a small town in western Poland, under the P1 project: Electronic Platform Collection, Analysis and Dissemination of Digital Medical Events. Depending on the results, the final project will be prepared to be implemented across Poland. There currently exists no nationwide system of Electronic Prescription Service in Poland. There is only a commercial e-Prescribing system produced by a commercial company, KAMSOFT information technology.

On-line adjudication of pharmacy claims is non-existent in the Polish health care system.

### Italy

Electronic prescriptions exchange, referred to as e-Prescribing, is used by 1% of general practitioner practices, which may come as a surprise since 86% of all Italian general practitioner practices use a computer and 71% of them are connected to the Internet [40].

Regional pilots for e-Prescribing started in Italy in 2002. In the Emilia-Romagna region, the programme called “SOLE – Online Healthcare” included electronic prescriptions management, whereas in Lombardy, a “Healthcare Extranet” (SISS) is the core project accumulating data on all the events taking place during treatment (from administration to prescription).

The present share of e-Prescribing compared to traditional written prescriptions is less than 20% [41].

In Italy, the transmission of electronic patient data has not been used extensively. Only 3% of Italian general practitioners exchange administrative data with health care providers. Online adjudication of pharmacy claims has not been well established, as only 1% of general practitioner practices exchange administrative information with reimbursers [40].

In Italy, the National Pharmaceutical Formulary is the competent authority to which pharmaceutical companies apply for reimbursement. Another agency (Italian Medicines Agency) is involved in the pricing and reimbursement procedures referring to novel drugs. Within Italian Medicines Agency, two committees act as advisory bodies [41].

Generic substitution has a negligible effect on the value of Italy's generics market. The share of generics in the pharmaceutical retail market (number of packs) is less than 20% [42]. In 2004, expenditure on off-patent drugs and semi-branded generics (in Italy generics are semi-branded – the name of the brand plus the name of the active substance) accounted for 10% and nearly 2% market share, respectively [42].

In Italy, unless the prescribers oppose substitution, pharmacists are allowed to substitute expensive original drugs with cheaper equivalents. However, Italian pharmacists are not financially motivated to support generics promotion [43]. Moreover, generics are not actively promoted by health insurance organisations because the drug prices are low [44]. The literature search confirmed that real-time adjudications have not yet been implemented in Italy.

### Germany

Germany has started the e-Health system implementation with security issues and is currently setting up a smartcard infrastructure. In 2005, the National eHealth strategy (“gematik”) defined e-prescription as the priority application. The national electronic prescribing record was initially planned to be mandatory, but is now voluntary, along with the electronic emergency data set and the personal electronic health record [45].

Various e-prescription pilot projects were pursued between 2003 and 2007. Pilot regions were Bochum-Essen (Nordrhein-Westfalen), Flensburg (Schleswig-Holstein), Heilbronn (Baden-Württemberg), Ingolstadt (Bayern), Löbau-Zittau (Sachsen), Trier (Rheinland-Pfalz), and Wolfsburg (Niedersachsen) [46].

In Germany, the basket of drugs to be reimbursed is determined by the Joint Federal Committee. Joint Federal Committee's membership includes hospitals, public health insurance companies and doctors’ federations. This organisation establishes the so-called negative lists and defines the financing conditions for medicines which are not eligible for obligatory prescription [33].

A widespread practice in German health care to substitute generic version of the same chemical agent is based on so-called rebate contracts [47]. Pharmacists, in order to obtain reimbursement from the statutory health insurance system (Gesetzliche Krankenversicherung), are required, whenever possible, to dispense a less expensive generic product for which a rebate contract was signed. Physicians can prevent drug substitution by crossing off “or the like” on the prescription formulary. This option was introduced in 2002, but the “rebate policy” came into being on 1 April 2007 [48]. Generic substitution has been also promoted by the Institute for Quality and Efficiency in Healthcare, with the aid of independent experts who assess medicinal products in terms of two criteria: therapeutic benefits and effectiveness in relation to equivalents [34]. The review found that real-time adjudications have not yet been implemented in Germany.

## Conclusions

According to our knowledge, this is the first study which investigates and reports on the use of Pharmacy Benefit Manager tools and techniques by organisations outside the USA. Pharmacy Benefit Managers, which were originally commissioned only to provide online adjudication of pharmacy claims, have developed a number of additional functions over time. Therefore, the Pharmacy Benefit Manager model – and the tools within this that have sought to better manage and coordinate the use of prescriptions – has proven to be highly successful in the USA, where these entities bring billions of dollars in savings to governments, private payers, and patients each year.

Our systematic review showed that most Pharmacy Benefit Manager tools have been already implemented, to a lesser or greater extent, by the European governments; however, their implementation is fragmented, not standardised, and varies across countries. On-line adjudication of pharmacy claims has not been routinely used in Europe. On the other hand, in the USA, this solution has proven to be highly efficient, time and cost saving. With the necessary software technology already in place, our main recommendation for governments would be to adopt an encouraging attitude towards this innovation in e-Health.

Some of the benefits arising from the implementation of Pharmacy Benefit Manager tools in InterQuality Partner countries are relatively easy to predict. For example, the elimination of false prescriptions alone would allow for considerable savings. Only a system which is operating in real time (online) is capable of preventing errors and abuse, and patients can avoid the negative effects of errors and mistakes occurring in the process of drug prescribing by doctors or drug dispensing by pharmacies. Significant savings in medical expenses from reducing the number of physician visits among chronically ill patients could be achieved through the use of automatic prescriptions renewing mechanism, with which chronically ill patients would be able to renew prescriptions without actually visiting the doctor in-person. The e-Prescribing is implemented as a fully functional service by only a few European countries. Intuitively, e-Prescribing adoption level should be consistent with the level of e-Health implementation. Although in 2010, only a few European Union member countries had implemented a fully operational primary care e-Prescribing system, it is currently actively being considered as an issue of national e-Health strategy in the European Union. Countries that have implemented complete e-Prescribing practice are Denmark, Sweden, Iceland and Estonia. e-Prescribing pilot projects are currently being tested in the Czech Republic, Italy, Finland and Poland. Regrettably, e-Prescribing, and not on-line adjudication of pharmacy claims, is sometimes considered to be the ultimate objective of e-Health implementation in the pharmaceutical sector.

Within the InterQuality Project, drug policy models based on Pharmacy Benefit Manager-type of organisations were recommended. Considering the possibilities of introducing Pharmacy Benefit Manager tools to the European markets, it seems that from the technical point of view, partial or complete implementation would be quite realistic in the foreseeable future. The progressing computerisation of the entire economy, including pharmacies and doctor's offices, combined with new information technology developments can encourage and support such investments. These favourable conditions have created new opportunities for Pharmacy Benefit Manager tools to be used throughout Europe. It should be emphasised that the European countries would not have to build up from scratch, but simply adopt, with some country-specific modifications, the well-proven Pharmacy Benefit Manager practices used for many years in the USA and Canada.

In the European Union, there is a strong drive to implement selected Pharmacy Benefit Manager tools and services of integrated care but little understanding that without proper institutional framework, they may not yield results comparable to those achieved in the USA. While most European Union Member States promote implementation of selected e-Health tools, like e-Prescribing, they are far from being integrated. Also outside Europe, e-Health tools are becoming increasingly popular. For example, Drug Utilization Review, which assures that patients receive appropriate drug therapy based on current medical guidelines, is even more developed in South Korea than in the USA. South Korea is considered to have one of the most innovative e-Health policies across the Organisation for Economic Co-operation and Development countries [49] However, South Korea's Drug Utilization Review is only a tool which helps ensure compliance with the drug policy, automatically eliminating only the most obvious abuses and threats to patient life and health. As a consequence, implementation of Drug Utilization Review in South Korea has yielded relatively low savings which are at the level of $10 million annually [50]. To increase savings, more attention should be paid to how to integrate the various e-Health tools to enhance their individual and collective benefit to the society. The European Union Member States should consider development of national platforms or an alternative means of integrating these tools. One of the models is the “pharmacy benefit management”, which provides an integrated package of cost-containment methods, implemented within a transparent institutional framework, powered by strong motivation of the agent. Without integration, individual e-Health tools will not work productively enough to achieve the common Member State goals related to cost and quality of pharmaceutical care. The key reasons for that are (i) diluted responsibilities, (ii) weak and sometimes conflicting motivation of different pharmaceutical sector institutions and health policy decision-makers, and (iii) weak enforcement of Pricing & Reimbursement regulations.

To summarise, in the USA, the health care system is based on the population criterion, in which privileges apply to the patient's right of access to medicines. In European Union Member State systems, the centre of gravity is the right of providers for public funding. The patient often continues to be treated as a “biological structure that generates revenue in the form of higher and higher copayments”. In the USA, in addition to facilitating access to medicines for those most in need, the use of population-based criteria reveals patients who generate the highest costs. This in turn allows the detection of errors in treatment, the patient visiting different doctors, unauthorised changes in pharmacotherapy or the weak patient's compliance. European Union systems usually lose track of such patients [51].

Considering the possibilities of introducing Pharmacy Benefit Managers in the European Union Member States, partial or complete implementation seems technically feasible in the foreseeable future, but comparable outcomes would not be realistically achievable without a comparable institutional framework.
